# Three-dimensional guidance including shape sensing of a stentgraft system for endovascular aneurysm repair

**DOI:** 10.1007/s11548-020-02167-2

**Published:** 2020-05-07

**Authors:** Sonja Jäckle, Verónica García-Vázquez, Tim Eixmann, Florian Matysiak, Felix von Haxthausen, Malte Maria Sieren, Hinnerk Schulz-Hildebrandt, Gereon Hüttmann, Floris Ernst, Markus Kleemann, Torben Pätz

**Affiliations:** 1 Fraunhofer MEVIS, Institute for Digital Medicine, Maria-Goeppert-Straße 3, 23562 Lübeck, Germany; 2 Institute for Robotics and Cognitive Systems, Universität zu Lübeck, Ratzeburger Allee 160, 23562 Lübeck, Germany; 3 Institute of Biomedical Optics, Universität zu Lübeck, Ratzeburger Allee 160, 23562 Lübeck, Germany; 4 Division of Vascular- and Endovascular Surgery, Department of Surgery, University Hospital Schleswig-Holstein, Ratzeburger Allee 160, 23562 Lübeck, Germany; 5 Department for Radiology and Nuclear Medicine, University Hospital Schleswig-Holstein, Ratzeburger Allee 160, 23562 Lübeck, Germany; 6 Medical Laser Center Lübeck GmbH, Peter-Monnik-Weg 4, 23562 Lübeck, Germany; 7 German Center for Lung Research (DZL) , Airway Research Center North, Wöhrendamm 80, 22927 Großhansdorf, Germany; 8 Fraunhofer MEVIS, Institute for Digital Medicine, Am Fallturm 1, 28359 Bremen, Germany

**Keywords:** Stentgraft system, Electromagnetic tracking system, Fiber Bragg gratings, Shape sensing, Endovascular navigation, Endovascular aneurysm repair

## Abstract

**Purpose:**

During endovascular aneurysm repair (EVAR) procedures, medical instruments are guided with two-dimensional (2D) fluoroscopy and conventional digital subtraction angiography. However, this requires X-ray exposure and contrast agent is used, and the depth information is missing. To overcome these drawbacks, a three-dimensional (3D) guidance approach based on tracking systems is introduced and evaluated.

**Methods:**

A multicore fiber with fiber Bragg gratings for shape sensing and three electromagnetic (EM) sensors for locating the shape were integrated into a stentgraft system. A model for obtaining the located shape of the first 38 cm of the stentgraft system with two EM sensors is introduced and compared with a method based on three EM sensors. Both methods were evaluated with a vessel phantom containing a 3D-printed vessel made of silicone and agar-agar simulating the surrounding tissue.

**Results:**

The evaluation of the guidance methods resulted in average errors from 1.35 to 2.43 mm and maximum errors from 3.04 to 6.30 mm using three EM sensors, and average errors from 1.57 to 2.64 mm and maximum errors from 2.79 to 6.27 mm using two EM sensors. Moreover, the videos made from the continuous measurements showed that a real-time guidance is possible with both approaches.

**Conclusion:**

The results showed that an accurate real-time guidance with two and three EM sensors is possible and that two EM sensors are already sufficient. Thus, the introduced 3D guidance method is promising to use it as navigation tool in EVAR procedures. Future work will focus on developing a method with less EM sensors and a detailed latency evaluation of the guidance method.

**Electronic supplementary material:**

The online version of this article (10.1007/s11548-020-02167-2) contains supplementary material, which is available to authorized users.

## Introduction

Aortic aneurysm is a local dilatation of the aorta with a diameter greater than 1.5 times of the normal size [11] and occurs most frequently in the abdominal part of the aorta. Untreated, abdominal aortic aneurysms (AAAs) enlarge over time and bare the risk of a rupture, which leads very fast to massive internal hemorrhages [6]. To lower the risk of vessel rupture, AAAs can be treated by implanting a stentgraft in the aneurysm region. This reduces the stress on the aneurysm wall. Usually, this is done with an endovascular aneurysm repair (EVAR) procedure [4], which is conducted minimal-invasively.

In EVAR procedures, fluoroscopy and conventional digital subtraction angiography (DSA) are the gold standard for guiding the medical instruments inside the patient’s body. The frame rate of fluoroscopy can be adapted from 1 fps up to 15 fps, and DSA imaging normally has a frame rate of 2 fps [5, 7]. A movement of the instrument is visible from 5 fps, but higher frame rates lead to higher exposures [2]. Thus, normally a frame rate around 7.5 fps is chosen for fluoroscopy [5, 7]. In addition, contrast agent is administered to show the current vessel volume. However, this guidance has several disadvantages. The patient and the physicians are exposed to X-rays, and contrast agent is potentially kidney damaging for the patient [17]. Moreover, the depth information is missing in the two-dimensional (2D) fluoroscopy and DSA images. This makes the navigation of the instruments challenging and can lead to prolonged procedure times. Ideally, the surgeon would like to have an accurate and real-time three-dimensional (3D) guidance without the need for X-rays and the administration of contrast agent. Previous studies have concluded that an accuracy of $<{5}\,\hbox {mm}$ is sufficient for most EVAR interventions, but for fenestrated EVAR, a higher accuracy is needed [3, 14]. Moreover, tracking systems provide a guidance with high frequencies (10 Hz and more). Thus, a 3D guidance based on tracking systems is preferable.

In the last years, several studies [12, 16, 19] used optical fibers with fiber Bragg gratings (FBGs) to provide shape sensing of medical instruments. FBGs are interference filters, which are inscribed into the core of an optical fiber and reflect a specific Bragg wavelength. Combining several FBGs at the same longitudinal position in different fiber cores as a FBG array allows to estimate curvature and direction angle, which can be used to reconstruct the shape of the fiber. This can be accomplished by gluing several fibers together or by using multicore fibers, which have FBGs inscribed in three or more cores of a single optical fiber [15].

Another tracking technology uses electromagnetic (EM) sensors, which can be used to track the position and orientation of medical instruments. The usage of EM tracking systems has been reported for various medical applications [8]. EM sensors can be easily integrated into medical tools such as needles, catheters or endoscopes. Furthermore, they do not need a line of sight to the base station, like optical tracking systems. Thus, they are suited to track instruments inside the human body. Usually, the current position and orientation (pose) of a tracked device are displayed in relation to preoperative data, such as computed tomography (CT) scans or 3D models of anatomical structures [3, 13, 14]. For this purpose, fiducial markers are placed on the patient during preoperative image acquisition. During navigation, these markers are placed at the same positions as in the preoperative scan and their positions in the EM space are determined by pointing them with the tip of an EM-tracked pointer. Then, the transformation from the intraoperative space to the preoperative space can be determined by means of using the marker coordinates in both spaces. As a result, the EM-tracked instruments can be visualized in the preoperative data.

Combining fiber optical shape sensing with EM tracking, the benefits of both tracking systems are used. With EM sensors, the current pose of the tracked instrument is obtained, whereas shape sensing shows the current bendings of the instrument, if it is hurting the vessel walls or if it is stuck somewhere. Shi et al. [18] reported a catheter including one EM sensor and an intravascular ultrasound probe at the tip, and an optical fiber with FBGs. However, no method for combining the reconstructed shape with the obtained EM sensor pose was introduced. Moreover, to our knowledge, no other groups have reported methods or experiments for the fusion of these two technologies.

In this work, we present a 3D guidance for a stentgraft system combining fiber optical shape sensing with EM tracking. A shape localization method based on two EM sensors is introduced and compared with an approach based on three EM sensors, which was already presented in [10]. Moreover, a realistic vessel phantom was built for evaluating both guidance methods. For this purpose, the stentgraft system was inserted with different insertion depths into the vessel phantom and the located shapes estimated by means of the guidance approach were compared with the shapes reconstructed from CT imaging. In addition, continuous measurements of the tracking systems were recorded and used for evaluation.

## Material and methods

### Stentgraft system

**Fig. 1:**
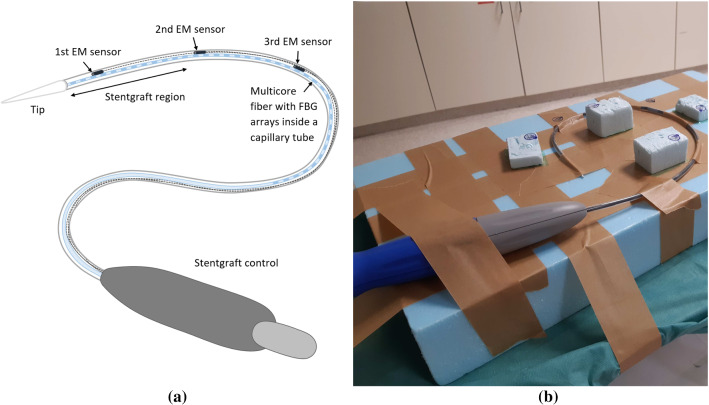
Sketch of the stentgraft system with integrated tracking systems (**a**) and setup for the calibration step (**b**)

A stentgraft system (Endurant®  II AAA, Medtronic, Dublin, Ireland) was disassembled, and the stentgraft was removed. Then, a multicore fiber (FBGS Technologies GmbH, Jena, Germany) inserted in a metallic capillary tube (400$\upmu$m diameter, AISI 304L) and three Aurora Micro 6-degree-of-freedom EM sensors (length: 9 mm, diameter: 0.8 mm; Northern Digital Inc., Waterloo, Canada) were integrated into the stentgraft system (Fig. 1a). The FBG arrays written into the optical fiber were not visible, but a fiber region of $40\, \hbox {cm}$ was marked by the manufacturer where the 38 FBG arrays are located. Thus, the first EM sensor was not placed exactly at the tip of the fiber but further inside to be sure that the sensor is within the shape sensing region. All EM sensors were fixed rigidly to the capillary tube and covered separately with a shrinkage tubing to protect them and their cables from damage. In addition, all EM sensors were placed at the front of the stentgraft system near the region where the stentgraft is placed.

### Tracking systems

The optical fiber was connected to a fanout and an interrogator (FBGS Technologies GmbH, Jena, Germany) to obtain the reflected wavelength of all FBGs. Then, the shape of the 38 cm shape sensing region of the fiber was reconstructed using the method explained in Jäckle et al. [9]. The resulting shape is represented as a point set

1 $$\begin{aligned} \hat{S} = \{S_0,\ldots , S_n\} \end{aligned}$$

with $n = 760$ and $|| S_i - S_{i+1} ||_2 = {0.5}\,{mm}$ distance in between, because the fiber has 38 cm shape sensing length and 20 interpolated positions were calculated per centimeter. Moreover, the direction vectors

2 $$\begin{aligned} \hat{D}^S = \{\hat{D}^S_0,\ldots , \hat{D}^S_n\} \end{aligned}$$

were computed for every shape sensing point during the shape reconstruction. Each element $\hat{D}^S_k$ is a three-element vector, which describes the direction of the shape for each point and can also be considered as a tangent vector of the reconstructed shape at each point.

The EM sensors were tracked using a Tabletop Field Generator (Northern Digital Inc., Waterloo, Canada). The current pose $\hat{P}_{k}^\mathrm{EM}$ of each EM sensor $k \in \{1,2,3\}$ in the EM space is defined as follows:

3 $$\begin{aligned} \hat{P}_{k}^\mathrm{EM} = \begin{pmatrix} \begin{array}{ccc} &{} &{} \\ &{} \hat{R}_{k}^\mathrm{EM} &{} \\ &{} &{} \\ \end{array} &{} \begin{array}{c} \\ \hat{T}_{k}^\mathrm{EM} \\ \\ \end{array} \\ \begin{array}{ccc} 0 &{} 0 &{} 0 \end{array}&1 \end{pmatrix} \end{aligned}$$

where $\hat{R}_{k}^\mathrm{EM}$ is a $3 \times 3$ matrix that contains the orientation information and $\hat{T}_{k}^\mathrm{EM}$ is a three-element vector with the position of the EM sensor tip. In addition, the direction vector $\hat{D}_{k}^\mathrm{EM}$ is given by $\hat{R}_{k}^\mathrm{EM}$ and corresponds to the third column of $\hat{R}_{k}^\mathrm{EM}$.

### Localization model

**Fig. 2:**
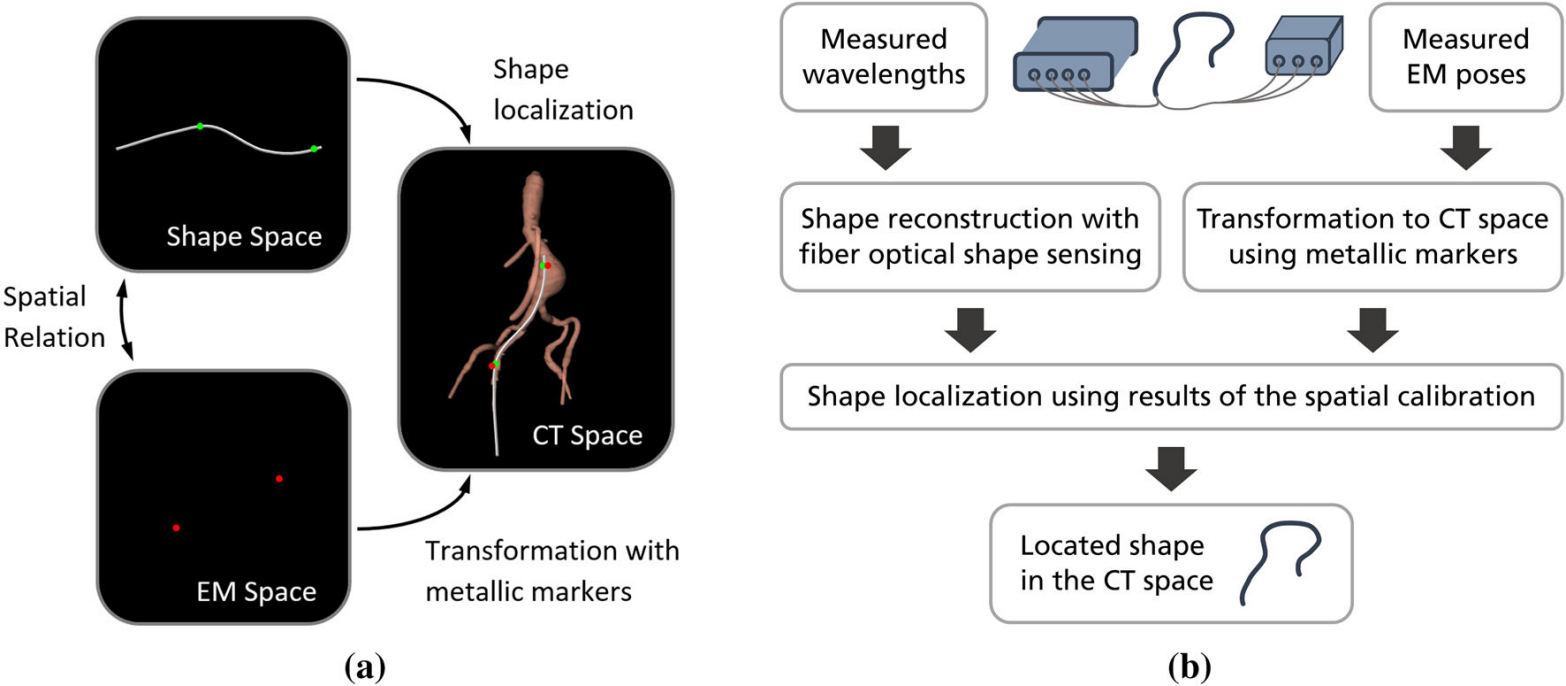
An illustration of all spaces and their relations when using two EM sensors (**a**) and a processing pipeline of the guidance based on the output data of the multicore fiber and the EM sensors (**b**)

For the calibration step and for the evaluation of the guidance methods, CT scans were made. To transform the EM sensor poses $\hat{P}_{k}^\mathrm{EM}$ from the EM space into the CT space $\hat{P}_{k}^\mathrm{CT}$, metallic markers were used in every measurement (Fig. 2a). A spatial calibration step was first made to find a correspondence between the shape $\hat{S}$ and the measured poses $\hat{P}_{k}^\mathrm{CT}$ of the EM sensors (Fig. 1b and Fig. 2a). In this step, the corresponding shape point $\hat{S}_{i_k}$ and the correction vector $\vec v_{k}$ for mapping each EM sensor position to its corresponding shape point were determined for each EM sensor. The calibration method was already introduced in detail in [10]. Afterwards, the shape $\hat{S}$ can be located in the CT space with the poses $\hat{P}_{k}^\mathrm{CT}$ of the EM sensors using the values obtained in the calibration. An overview of all processing steps is given in Fig. 2b. In the following subsections, the shape localization methods with three and two EM sensors are explained.

#### Three EM sensors

With the data from the tracking systems, the shape $\hat{S}$ was reconstructed in shape space and the measured EM poses $\hat{P}_{k}^\mathrm{EM} (k \in \{1,2,3\})$ in the EM space were obtained. Using the values obtained in the spatial calibration step, the shape points in the shape sensing space

4 $$\begin{aligned} \{\hat{S}_{i_1},\hat{S}_{i_2},\hat{S}_{i_3}\} \end{aligned}$$

and their corresponding points in the CT space

5 $$\begin{aligned} \{\hat{T}_{1}^\mathrm{CT}+\vec v_{1},\hat{T}_{2}^\mathrm{CT}+\vec v_{2},\hat{T}_{3}^\mathrm{CT}+\vec v_{3}\} \end{aligned}$$

can be determined. Using these two point sets, a rigid transformation was computed [1]. This transformation can be used to locate the reconstructed shape $\hat{S}$ in the CT space.

#### Two EM sensors

In this case, the position and orientation of the first and third EM sensors are used. Moreover, the direction information $\hat{D}^S$ along the shape is obtained during shape reconstruction. Using the information of the spatial calibration, two shape points in the shape sensing space

6 $$\begin{aligned} \{\hat{S}_{i_1},\hat{S}_{i_3}\} \end{aligned}$$

and their corresponding points in the CT space

7 $$\begin{aligned} \{\hat{T}_{1}^\mathrm{CT}+\vec v_{1},\hat{T}_{3}^\mathrm{CT}+\vec v_{3}\} \end{aligned}$$

were obtained. However, two points are not sufficient to determine a rigid transformation. For this reason, two additional points were generated by adding the direction vector with 10 mm length. The directions of the shape points $\hat{D}^S_{i_k}$ were computed during the shape reconstruction, and the direction of the EM sensor $\hat{D}^\mathrm{CT}_{k}$ corresponded to the third column of $\hat{R}_{k}^\mathrm{CT}$. Then, four shape points in the shape sensing space

8 $$\begin{aligned} \{\hat{S}_{i_1}, \hat{S}_{i_1} + 10 \cdot \hat{D}^S_{i_1}, \hat{S}_{i_3}, \hat{S}_{i_3} + 10 \cdot \hat{D}^S_{i_3}\} \end{aligned}$$

and their corresponding points in the CT space

9 $$\begin{aligned}&\{\hat{T}_{1}^\mathrm{CT}+\vec v_{1}, \hat{T}_{1}^\mathrm{CT} + \vec v_{1} \nonumber \\&\quad + 10 \cdot \hat{D}^\mathrm{CT}_{1}, \hat{T}_{3}^\mathrm{CT} + \vec v_{3}, \hat{T}_{3}^\mathrm{CT} + \vec v_{3} + 10 \cdot \hat{D}^\mathrm{CT}_{3}\} \end{aligned}$$

were determined. These two point sets were used to calculate the rigid transformation [1] for locating the reconstructed shape $\hat{S}$ in the CT space.

### Evaluation

#### Vessel phantom

**Fig. 3:**
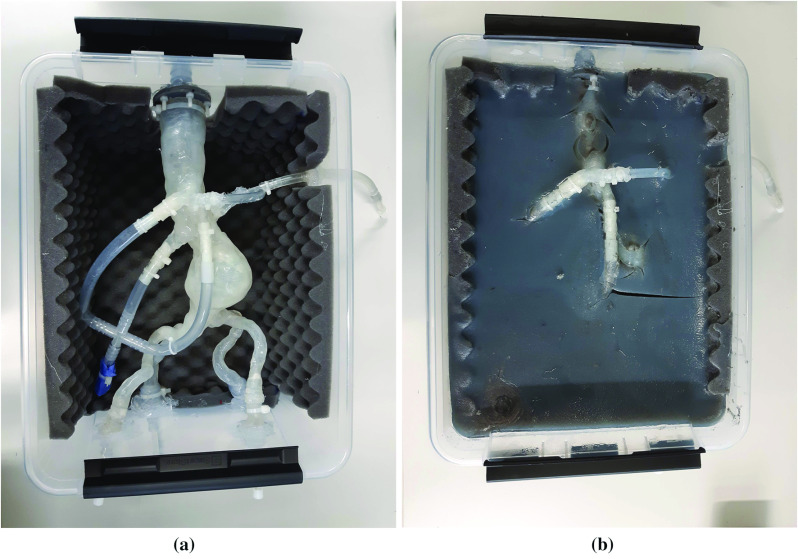
The vessel phantom without (**a**) and with (**b**) agar–agar

A 3D-printed vessel made of silicone and built from patient data (HumanX GmbH, Wildau, Germany) was integrated into a plastic container with size: $40\,\hbox {cm} \times 30\,\hbox {cm} \times 19\,\hbox {cm}$ (Fig. 3a). For this purpose, access points were created and the sides of the container were covered with foam. Afterwards, the vessel was inserted and the iliac arteries were fixed with silicone to avoid leakages. In the second step, an artificial surrounding tissue was made with agar–agar. ${600}\,{g}$ agar–agar were stirred in ${11,3}\,{{\hbox {l}}}$ water while heating it up to ${63}\,{^{\circ }\mathrm {C}}$. When the agar–agar was dissolved, ${700}\,{{\hbox {ml}}}$ glycerol and ${40}\,{\mathrm{g}}$ graphite were included in the mixture. Then, the phantom was cooled down ${16}\,{\mathrm{h}}$ at room temperature and afterwards ${7}\,{\mathrm{h}}$ in the fridge. The resulting phantom is shown in Fig. 3b.

#### Experiments

For the spatial calibration step, the stentgraft system was fixed in a bow shape to a rigid foam placed on a CT table and six metallic markers (SL10, diameter: 1 mm; The Suremark Company, California, USA) were placed at different heights around the stentgraft system to transform the poses of the EM sensors into those in the CT space (Fig. 1b).

For the evaluation of the catheter guidance methods, the vessel phantom was placed and fixed on the CT table and five metallic markers were placed on the plastic box of the phantom. For introducing the stentgraft system, first a soft guide wire was inserted, then a standard catheter was pushed over and the soft guide wire was replaced with a stiff guide wire. After that, the catheter was removed. Finally, the stentgraft system was inserted into the phantom, moved to the aneurysm and pulled back in $5\,\hbox {cm}$ steps using the stiff guide wire. Ultrasound gel was used to facilitate the insertion of the wires, catheter and the stentgraft system. Moreover, continuous measurements of the optical fiber and the EM sensors were made while moving the stentgraft system to the aneurysm. The data from both tracking systems were obtained at a frequency of 10 Hz.

#### Evaluation measures

For the spatial calibration step and for each insertion depth of the stentgraft system, the data from the optical fiber and the EM sensors were measured before and after the acquisition of a CT scan (which was used to obtain the ground truth) in order to evaluate the stability of the whole setup.

Each CT study was made with a Siemens SOMATOM Definition AS+ scanner. In the spatial calibration step, the scan was acquired with the parameters: voltage of 120 KVp, exposure of 109 mAs, image size of ${512 \times 512 \times 733}$ and voxel size of  $0.51 \times 0.51 \times {0.40}\,{\mathrm{mm}}$. For the evaluation experiments, the following parameters were used: voltage of 120 KVp, exposure of 180 mAs, image size of ${512 \times 512 \times 1156}$ and voxel size of $0.70 \times 0.70 \times {0.60}$ mm.

The reconstructed shapes before and after each CT acquisition were aligned by means of a point-based registration [1] and compared to evaluate the shape movement. In addition, the maximal position change $c_{\text {p}}$ and the maximal orientation angle change $c_{\text {o}}$ of the EM sensors before and after each CT acquisition were calculated.

Afterwards, the shapes of the stentgraft system were segmented, the EM sensor positions were obtained manually from each CT scan and both were used as ground truth for the comparison with the estimated measurements. The positions of the metallic markers, which were used for transforming the EM sensor positions into the CT space, were determined semiautomatically. For this, each marker was segmented by thresholding and the centroid of each marker segmentation was calculated, which results in a subvoxel precision for the marker localization.

For evaluation, the reconstructed shape was aligned with the ground truth shape by means of point-based registration [1]. The average and maximum errors defined as

10 $$\begin{aligned} e_{\text {avg}}&:= \frac{1}{m + 1}\sum _{i=0}^{m} \Vert x_i - x^{\text {gt}}_i \Vert _2 \text { and }\nonumber \\ e_{\text {max}}&:= \max ( \Vert x_0 - x_0^{\text {gt}} \Vert _2, \ldots , \Vert x_m - x_m^{\text {gt}} \Vert _2) \end{aligned}$$

where $x_0, \ldots , x_m$ are the estimated points and $x_0^{gt}, \dots , x_m^{gt}$ are the ground truth points were calculated. For the evaluation of the shape movement and the reconstructed and located shapes, the points were compared every $10 \, \text {mm}$ along the shape.

## Results and discussion

**Table 1:** The average and maximum shape movements ($e_{\text {avg}}$ and $e_{\text {max}}$ in mm), the movement of the EM sensors $c_{\text {p}}$ (in mm) and $c_{\text {o}}$ (in degrees), and the measured errors $e_{\text {avg}}$ and $e_{\text {max}}$ (in mm) of the whole reconstructed shape, the EM sensor positions and the whole located shape using three or two EM sensors for the calibration step and the stentgraft system inserted at three different depths into the vessel phantom

	Shape movement		EM sensor movement		Reconstructed shape		EM sensor positions		Located shape (3 EM sensors)		Located shape (2 EM sensors)	
Shape	Error											
	$e_{\text {avg}}$	$e_{\text {max}}$	$c_{\text {p}}$	$c_{\text {o}}$	$e_{\text {avg}}$	$e_{\text {max}}$	$e_{\text {avg}}$	$e_{\text {max}}$	$e_{\text {avg}}$	$e_{\text {max}}$	$e_{\text {avg}}$	$e_{\text {max}}$
Calibration	0.04	0.16	0.04	0.08	0.69	1.93	0.96	1.30	2.01	6.30	1.57	3.57
22 cm inside	0.03	0.12	0.03	0.06	0.48	1.04	1.07	1.44	2.43	3.04	2.64	3.22
17 cm inside	0.02	0.06	0.04	0.13	0.87	1.55	1.48	1.85	1.47	3.54	1.70	2.79
12 cm inside	0.03	0.09	0.06	0.11	0.60	1.42	1.31	1.70	1.35	3.49	2.47	6.27

The results of the spatial calibration step and the measurements of the stentgraft system inserted at three different depths into the vessel phantom are shown in Table 1. The movements of the EM sensors and the reconstructed shapes before and after each CT acquisition were very low in all experiments. This indicates that the EM sensors and the optical fiber were fixed very well inside the stentgraft system and that their setup before and after each CT acquisition should match. Thus, the measured shapes and EM sensor positions could be compared to the segmented ones from the CT (reference) for evaluating the accuracy of both tracking systems.

For all experiments, the shapes were reconstructed accurately (${e_{\text {avg}} < {0.9}\,\hbox {mm}}$ and $e_{\text {max}} <{2.0}\,\hbox {mm}$). The accuracies are comparable to previous experiments [9, 10] but higher than those reported in [12] (shape reconstruction of a catheter containing four multicore fibers and with a length of 11.8 cm, maximum error: 1.05 mm). Nevertheless, our reconstructed shapes with only one multicore fiber were longer (specifically, 38 cm), more flexible and complex than the evaluated shapes in [12]. In addition, the EM sensors’ positions were measured accurately (${e_{\text {avg}} <{1.5}\,\mathrm{mm}}$ and ${e_{\text {max}} <{1.9}\,\hbox {mm}}$) and the measured errors were comparable to those reported in [3, 13, 14] (in each study, average error: 1.20 mm/1.30 mm/1.28 mm and maximum error: 1.70 mm/1.89 mm/2.98 mm).

In the spatial calibration step, the corresponding shape points with the indices $i_1 = 699,\, i_2 = 498,\, i_3 = 299$ located at 34.95 cm, 24.90 cm and 14.95 cm along the shape sensing region of the multicore fiber, respectively, and the correction vectors $\vec v_1, \vec v_2$ and $\vec v_3$ were determined. The EM sensors were placed with approximately $10\,\hbox {cm}$ between each other at the front of the stentgraft system and within the shape sensing region to ensure a high accuracy for the first 20 cm of the stentgraft system because the stentgraft is integrated in this region.

**Fig. 4:**
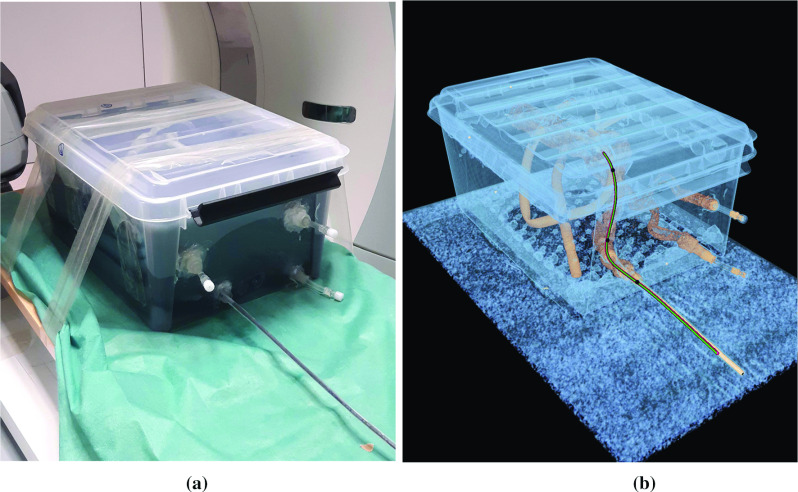
Stentgraft system inserted into the vessel phantom (**a**) and its corresponding CT scan (**b**) of the deepest insertion with the located shapes using three EM sensors (red) and two EM sensors (green), and the EM sensor positions (black)

For the evaluation of the catheter guidance methods, the guide wires, the catheter and the stentgraft system were inserted into the right internal iliac artery of the vessel phantom, as shown in Fig. 4a. The tracking data of the stentgraft system were measured at 22 cm, 17 cm and 12 cm insertion depth. The measured average and maximum errors of the shapes located with three or two EM sensors are generally higher than the measured errors of the reconstructed shape and the EM sensor positions separately (Table 1). The reason for this is that both the EM sensor errors and the shape errors influence the resulting located shape. In comparison with a previous study [10], the errors of the shapes located with three EM sensors are higher. However, the results are not completely comparable because in [10] the EM sensors were located at the tip, middle and end of the 38 cm shape sensing region of a catheter. In this work, the three EM sensors were positioned at the front of the stentgraft system (approximately 20 cm). The accuracies of the located shaped inside the vessel phantom (Table 2) indicate that the highest errors are usually at the back of stentgraft system and that its front shape is located generally more accurately.

Moreover, two videos about the guidance based on three and two EM sensors were made using the continuous measurements (see electronic supplementary materials attached to the paper: three EM sensors = material_1, two EM sensors = material_2). The recorded tracking data were replayed half as fast as recorded specifically with 5 Hz, to clearly see the stentgraft system movements, since it was moved quickly. In most of the time, the shape of the stentgraft system is inside the vessel system, but in the last part of the videos the back is not always inside. In both videos, the front of the stentgraft system is located at approximately the same position and the highest differences are at the back. The reason for this is that the back of the stentgraft system contains no EM sensors. Moreover, the shape is located fluently and no lagging can be observed in both videos.

**Table 2:** Measured errors $e_{\text {avg}}$ and $e_{\text {max}}$ (in mm) of the located shape inside the vessel phantom

	Located shape (3 EM sensors)		Located shape (2 EM sensors)	
Shape	Error			
	$e_{\text {avg}}$	$e_{\text {max}}$	$e_{\text {avg}}$	$e_{\text {max}}$
22 cm inside	2.38	3.04	2.50	3.01
17 cm inside	0.79	2.00	1.46	1.87
12 cm inside	1.78	3.49	2.17	3.19

Comparing the approaches to locate the shape with three or two EM sensors, the errors of the located shapes (Tables 1 and 2) are comparable. This is also visible in Fig. 4b. The shape located with two EM sensors is positioned a little further to the left than that located with three EM sensors at the part outside of the phantom, but the shapes inside the vessel are indistinguishable. Moreover, the attached videos about the navigated stentgraft system show that a fluent guidance is possible with both approaches. Thus, both methods enable a comparable guidance of the stentgraft system. In summary, two EM sensors are sufficient for determining an accurately located shape.

Theoretically, one EM sensor should be sufficient for the shape localization, since it provides the pose information. In this case, an exact calibration between the EM sensor and the optical fiber is needed. However, we observed that this is not possible, since the orientation of a fixed fiber is not constant over time or even not uniquely defined in straight parts of the reconstructed shape (data not shown). Thus, locating the shape with one EM sensor is not easily possible.

As described in Introduction, the clinical requirements for a guidance in EVAR procedures are an accuracy $<{5}\,\hbox {mm}$ and a frequency of $>{7.5}\ \hbox {Hz}$. Thus, the results showed that the introduced 3D guidance approach could be used in EVAR procedures.

However, when using the tracked stentgraft system in a real EVAR procedure, the errors of the EM sensors and thus the located shape are expected to be higher. EM sensors are susceptible to interference with metallic and electronic medical instruments, which results in decreasing accuracy of the measured EM sensor poses [8]. Moreover, the metallic markers have to be placed exactly at the same positions on the patient as in the preoperative CT scan to transform the measured EM poses into the CT space. Error sources of that registration are different patient positioning during the intervention than that during the acquisition of the preoperative CT scan, tissue deformation, breathing and arterial pulsation. In addition, EM sensors have the limitation that they can only be tracked in the measurement volume of the field generator. Thus, the amount of used EM sensors limits the range where the instrument can be tracked.

On the other hand, shape sensing using multicore fibers with FBGs has also limitations. Due to their physical properties, bending diameters less than $2\,\hbox {cm}$ cannot be measured and the fiber might break. For this reason, this technology cannot be integrated into very flexible instruments, such as soft guide wires. Moreover, dynamical twists might not be detected using a multicore fiber with parallel-positioned FBGs. Fibers with other geometrically arranged FBGs, such as helical wrapped FBGs [20], can measure twists of the optical fiber. However, using the introduced stentgraft system, no dynamic twist or bending diameters less than $2\,\hbox {cm}$ are expected during usage.

## Conclusion

This study introduced and compared two different methods for determining the located shape of a stentgraft system using tracking systems. A multicore fiber and three EM sensors were integrated into the front of a stentgraft system. Furthermore, a phantom with a 3D-printed vessel from patient data and an artificial surrounding tissue was built to evaluate those methods. After a calibration step, the tracked stentgraft system was inserted into the phantom, measurements were taken at different insertion depths and continuous data were recorded.

The evaluation of both methods showed that the shapes located with three EM sensors ($e_\mathrm{avg} \approx$ 1.35 to 2.43  mm and $e_\mathrm{max} \approx$ 3.04 to 6.30 mm) are comparable to the shapes located with two EM sensors ($e_\mathrm{avg} \approx$ 1.57 to 2.64 mm and $e_\mathrm{max} \approx$ 2.79 to 6.27 mm). Moreover, a comparable fluent guidance is possible with both approaches. Thus, the usage of two EM sensors is sufficient for determining accurate located shapes of the stentgraft system.

For using these guidance methods in EVAR procedures, an accuracy of $< {5}\,\hbox {mm}$ and a frequency of $>{7.5}\ \hbox {Hz}$ are required. Thus, the results of the guidance method evaluated with a realistic phantom are promising for using this tracking method as navigation support during an EVAR procedure. Moreover, this approach can be also applied for the navigation of medical instruments in other interventions such as bronchoscopy or endoscopy. Furthermore, the combination of a tracking-based guidance with robotic systems is possible. The tracking systems can give the robotic system a direct feedback about the current state of the inserted instrument, and thus, a combination of both can enable a fully autonomous navigation.

Future work will focus on a further reduction of the necessary EM sensors for an accurate shape localization to overcome the limitation of the EM measurement volume. In this case, further restrictions, such as that the reconstructed shape should be inside the vessel, are necessary to locate the shape more accurately. In addition, the latency of the guidance methods will be evaluated.

## Electronic supplementary material

Below is the link to the electronic supplementary material.

Supplementary material 1 (mp4 921 KB)

Supplementary material 2 (mp4 920 KB)
